# Relationship between Thrombosis Risk Factors, Clinical Symptoms, and Laboratory Findings with Pulmonary Embolism Diagnosis; a Cross-Sectional Study

**Published:** 2019-07-23

**Authors:** Rama Bozorgmehr, Mehdi Pishgahi, Pegah Mohaghegh, Marziye Bayat, Parastou Khodadadi, Ahmadreza Ghafori

**Affiliations:** 1 Internal Medicine Department, Clinical Research Development Unit, Shohadaye Tajrish Hospital, Shahid Beheshti University of Medical Science, Tehran, Iran; 2 Cardiology Department, Shohadaye Tajrish Hospital, Shahid Beheshti University of Medical Sciences, Tehran, Iran; 3 Department of Community and Preventive Medicine, School of Medicine, Arak University of Medical Science, Arak, Iran

**Keywords:** Pulmonary embolism, Computed Tomography Angiography, diagnosis, risk factors, signs and symptoms, symptom assessment

## Abstract

**Introduction:**

Pulmonary embolism (PE) is a potentially life threatening disease, accurate and timely diagnosis of which is still a challenge that physicians face. This study was designed with the aim of evaluating the relationship between thrombosis risk factors, clinical symptoms, and laboratory findings with the presence or absence of PE.

**Methods:**

The present retrospective cross-sectional study was performed on patients with suspected pulmonary embolism who were hospitalized in different departments of Shohadaye Tajrish Hospital, Tehran, Iran, during 1 year. All patients underwent computed tomography pulmonary angiography (CTPA) and then thrombosis risk factors, clinical symptoms, and laboratory findings of confirmed PE cases with CTPA were compared with others.

**Results:**

188 patients with the mean age of 61.91 ± 18.25 (20 – 101) years were studied (54.8% male). Based on Wells' score, 32 (17.2%) patients were in the low risk group, 145 (78.0%) were in the moderate risk group, and 9 (4.8%) patients were classified in the high risk group for developing PE. CTPA findings confirmed PE diagnosis for 60 (31.7%) patients (6.7% high risk, 75.0% moderate risk, 18.3% low risk). D-dimer test was only ordered for 27 patients, 25 (92.6%) of which were positive. Among the patients with positive D-dimer, 18 (72.0%) cases had negative CTPA. Inactivity (57.4%), hypertension (32.8%), and history of cancer (29.5%) were the most common risk factors of thrombosis in patients with PE. In addition, shortness of breath (60.1%) and tachypnea (11.1%) were the most common clinical findings among patients with PE. There was no significant difference between the patients with PE diagnosis and others regarding mean age (p = 0.560), sex distribution (p = 0.438), and type of thrombosis risk factors (p > 0.05), hospitalization department (p = 0.757), Wells’ score (p = 0.665), electrocardiography findings, or blood gas analyses.

**Conclusion:**

Although attention to thrombosis risk factors, clinical symptoms, and laboratory findings, can be helpful in screening patients with suspected PE, considering the ability of CT scan in confirming or ruling out other possible differential diagnoses, it seems that a revision should be done to lower the threshold of ordering this diagnostic modality for suspected cases.

## Introduction

Pulmonary embolism (PE) is a common and potentially life threatening disease. In population-based studies, incidence rate of PE adjusted for age and sex has been estimated to be 21 to 69 patients in each 100000 population per year (1, 2). On the other hand, PE is the cause of 1.3% to 10% of all in-hospital deaths (3-5). A relatively considerable number of PE and deep vein thrombosis (DVT) cases currently occur in orthopedics, gynecology, surgery, urology, and … departments, which seem preventable. Due to the high rate of mortality due to PE (up to 30%), timely initiation of proper treatment can lead to a decrease in mortality by up to 8% (6). Accurate and timely diagnosis of PE is still a challenge faced by physicians. Due to presence of a wide range of non-specific symptoms such as chest pain, hemoptysis, and dyspnea, accurate and rapid diagnosis of PE can be mistaken with other differential diagnoses. In 75% of patients with suspected primary symptoms, PE is confirmed in final evaluations (7). The results of a meta-analysis showed that clinical symptoms alone have 85% sensitivity and 51% specificity for diagnosis of this disease (8). Therefore, having strong clinical suspicion is vital for diagnosis of the disease and not missing true cases. Among diagnostic methods, computed tomography pulmonary angiography (CTPA), is the best imaging method and is necessary for definite diagnosis (9). Of course, this valuable diagnostic modality is not available everywhere and in addition to imposing costs on the patient and healthcare system, it is also associated with unavoidable side effects such as radiation and probability of reaction to radio contrast agent. 

Currently simple and available tools, such as arterial blood gas analysis, electrocardiography (ECG) findings, and clinical decision rules such as Wells’ score have been considered for help in triage of suspected cases. However, a consensus regarding their diagnostic value and screening performance characteristics for the mentioned purpose has not been reached, yet. 

Considering the afore-mentioned points, the present study has been designed with the aim of evaluating the relationship between risk factors, clinical symptoms, and laboratory findings with the CTPA confirmed PE. 

## Methods

***Study design and setting***

In the present retrospective cross-sectional study, all patients with suspected PE who were hospitalized in different departments of Shohadaye Tajrish Hospital, Tehran, Iran, during 1 year and underwent CTPA were evaluated. Protocol of the study was approved by the research council and ethics in research committee of Shahid Beheshti University of Medical Sciences (Ethics code: IR.SBMU.RETECH.REC.1397.546). The checklist for data gathering was filled out anonymously using medical profile codes for each patient with suspected PE.

***Participants***

Patients suspected to PE hospitalized in different departments (internal medicine, surgery, intensive care, oncology, and …) of the mentioned hospital who had undergone CTPA and their clinical profile was available were included in the study. No age or sex limitation was considered for inclusion in the study. Patients with missing data in their clinical profile were excluded from the study.

***Data gathering***

For gathering data, a checklist consisting of demographic data (age, sex), known risk factors of thrombosis (history of cancer, obesity, trauma, and …), clinical findings (shortness of breath, tachycardia, tachypnea, hemoptysis, and …), laboratory parameters (D-dimer, blood gas analysis), the result of CTPA, electrocardiographic (ECG) manifestations, risk of developing PE based on Wells’ score (high risk, low risk, and moderate risk), as well as outcome (death and survival) was filled out for all patients by referring to their clinical profile. Checklists were anonymous and filled using the profile code for each patient. A trained intern was responsible for data gathering under direct supervision of an internal medicine specialist.

All CTPAs had been evaluated and interpreted by the radiology department of the hospital regarding presence or absence of PE.

***Statistical analysis***

Patients’ data were entered to SPSS version 23 statistical software. Quantitative data were described using mean and standard deviation (SD) and qualitative data were described using frequency and percentage. To determine if the data was normal or not, Kolmogorov-Smirnov test was used. If quantitative data had normal distribution, independent t-test was used; and otherwise, non-parametric equivalent test (Mann-Whitney U test) was applied. To evaluate the correlation between qualitative variables, chi-square and Fisher’s exact tests were used. All analyses were performed with an alpha error of 5%.

**Table 1:** Baseline characteristics of the studied patients

**Variables **	**Total (n = 188)**	**Confirmed PE**		**P value**
		**No (n = 127)**	**Yes (n = 60)**	
**Age (years)**				
Mean ± SD	61.91 ± 18.25	61.39 ± 18.71	63.02 ± 17.37	0.56
**Sex (%)**				
Male	85 (45.5)	60 (47.2)	25 (41.0)	0.438
Female	103 (54.8)	67 (52.8)	36 (59.0)	
**Thrombosis risk factors**				
History of DVT	6 (3.2)	5 (3.9)	1 (1.6)	0.666
Obesity	5 (2.7)	3 (2.4)	2 (3.3)	0.660
History of cancer	53 (28.2)	35 (27.6)	18 (29.5)	0.863
Hypertension	65 (34.6)	45 (35.4)	20 (32.8)	0.721
OCP or HRT	2 (1.1)	1 (0.8)	1 (1.6)	0.545
COPD	7 (3.7)	4 (3.1)	3 (4.9)	0.684
Pregnancy	1 (0.5)	1 (0.8)	0 (0)	1.000
Recent surgery	33 (17.6)	24 (18.9)	9 (14.8)	0.544
Thrombophilia	0 (0)	0 (0)	0 (0)	NA
Trauma history	31 (16.5)	18 (14.2)	13 (21.3)	0.217
Inactivity	103 (54.8)	68 (53.5)	35 (57.4)	0.621
Recent travel	1 (0.5)	0 (0)	1 (1.6)	0.148
**Clinical symptoms**				
Chest pain	15 (7.9)	10 (7.8)	5 (8.3)	0.891
Dyspnea	112 (59.6)	76 (58.9)	36(60.1)	0.888
Tachycardia	14 (7.5)	9 (7.0)	5 (8.3)	0.740
Tachypnea	20 (10.7)	13 (10.1)	7 (11.7)	0.741
Hemoptysis	2 (1.1%)	2 (1.6)	0 (0)	0.323
**Wells’ Score**				
High risk	9 (4.8)	5 (55.6)	4 (44.4)	0.665
Moderate risk	146 (78.1)	101 (69.2)	45 (30.8)	
Low risk	32 (17.1)	21 (65.6)	11 (34.4)	
**Outcome**				
Survived	139 (73.9)	94 (74.0)	45 (73.8)	0.971
Not survived	49 (26.1)	33 (26.0)	16 (26.2)	

Data are presented as mean ± standard deviation (SD) or number (%). NA: not applicable. DVT: deep vein thrombosis, OCP: oral contraceptive, HRT: hormone replacement therapy; COPD: chronic obstructive pulmonary disease; PE: pulmonary embolism.

**Table 2:** Electrocardiogram (ECG) manifestations and blood gas analysis of the studied subjects

**Variables **	**Total (n = 188)**	**Confirmed PE**		**P value**
		**No (n = 127)**	**Yes (n = 60)**	
**ECG manifestation **				
Sinus Tachycardia	105 (55.9)	73 (57.5)	32 (52.5)	0.516
Atrial fibrillation	10 (5.3)	8 (6.3)	2 (3.3)	0.338
S1Q3T3 pattern	48 (25.5)	32(25.2)	16 (26.2)	0.879
T inversion in V1-3	34(18.1)	20(15.7)	14 (23)	0.230
Right bundle brunch block	20 (10.6)	13 (10.2)	7 (11.5)	0.796
**Arterial blood gas analyses **				
O_2_ saturation (%)	84.97 ± 15.45	85.66 ± 15.73	83.54 ± 14.86	0.131
pH	7.40 ± 0.08	7.40 ± 0.09	7.38 ± 0.06	0.145
PCO_2_ (mmHg)	46.32 ± 12.98	49.56 ± 15.70	39.59 ± 2.68	0.893
HCO_3_ (mmHg)	22.62 ± 7.43	22.55 ± 6.41	22.75 ± 9.27	0865
PO_2_ (mmHg)	66.04 ± 36.80	69.42 ± 38.97	58.93 ± 38.95	0.162

Data are presented as mean ± standard deviation (SD) or number (%).PE: pulmonary embolism.

## Results

188 patients with the mean age of 61.91 ± 18.25 (20 – 101) years were studied (54.8% male). Table 1 shows the baseline characteristics of the studied patients. The highest number of cases with suspected PE belonged to internal medicine (33.9%), surgery (30.5%), and intensive care (14.1%) departments. Mean Wells’ score of the patients was 3.88 ± 1.15 (0 – 9). Based on Wells’ score, 32 (17.2%) patients were in the low risk group, 145 (78.0%) were in the moderate risk, and 9 (4.8%) patients were classified in the high risk group for developing PE. 

CTPA findings confirmed PE diagnosis for 60 (31.7%) patients (6.7% high risk, 75.0% moderate risk, 18.3% low risk).

D-dimer test was only ordered for 27 patients, 25 (92.6%) of which were positive. Among the patients with positive D-dimer, 18 (72.0%) cases had negative CTPA.

Inactivity (57.4%), hypertension (32.8%), and history of cancer (29.5%) were the most common risk factors of thrombosis incidence in patients with PE. In addition, shortness of breath (60.1%) and tachypnea (11.1%) were the most common clinical findings among patients with PE. There was no significant difference between the patients with PE diagnosis and other patients regarding mean age (p = 0.560), sex distribution (p = 0.438), type of thrombosis risk factors (p > 0.05), hospitalization department (p = 0.757), and Wells’ score (p = 0.665).

Table 2 depicts ECG findings and blood gas analyses of the studied patients. Sinus tachycardia (52.5%), S1Q3T3 pattern (26.2%), and T inversion (23.0%) were the most common findings in the ECGs of patients with PE. However, there was no significant difference between the PE group and other patients regarding ECG findings and blood gas analyses.

Sensitivity, specificity, positive and negative predictive values of Wells’ score with 95% confidence interval (CI) in these patients were 31.61% (95% CI: 24.51 – 39.63), 65.62% (95% CI: 46.77 – 80.82), 81.86 (95% CI: 69.14 – 90.06), and 16.53 (95% CI: 10.75 – 24.39), respectively. The accuracy of Wells’ score based on area under the receiver operating characteristic (ROC) curve was estimated as 0.509 (95% CI: 0.420 – 0.598).

## Discussion

Based on the results of the present study, the prevalence of PE among the studied patients was estimated to be about 32%. There was no significant difference between patients with confirmed diagnosis of PE and other suspected cases regarding age, sex, clinical symptoms, laboratory findings, ECG manifestations, hospitalization department, and Wells’ score.

In a meta-analysis, Moores et al.‌ reported the prevalence of PE as 15% - 37% based on the results of CTPA (8). This rate was estimated as 17.1% in the study by Abidi et al. and about 60% in the study by Sodhi et al. (10, 11).

In the present study, no significant statistical correlation was observed between PE diagnosis and risk factors, clinical symptoms, or laboratory findings. Based on ESC guidelines on the diagnosis and management of acute PE, 2014 in evaluating differential diagnoses of PE, no symptom alone can rule out this diagnosis and clinical symptoms such as shortness of breath, tachycardia, tachypnea, chest pain, and hemoptysis are non-specific for diagnosis of PE (12).

In the preset study, the most common cardiac findings in the ECGs of the patients with PE were sinus tachycardia, followed by S1Q3T3 pattern and T inversion. In our evaluation, there was no statistically significant difference between patients with and without PE regarding ECG findings. Numerous studies have evaluated the use of ECG and its ability to diagnose PE (13, 14). In the study by Sinha et al. sinus tachycardia, S1Q3T3 pattern, atrial tachyarrhythmia, presence of Q wave in lead III and Q3T3 pattern were among the findings related with PE. Sinha et al. expressed that these findings are generally non-specific and mostly change throughout time and may worsen the outcome of PE in some cases (14). They reported that classic S1Q3T3 pattern lacked the required specificity in diagnosis and prognosis determination of PE. However, a study performed by Bircan et al. showed that after confirming diagnosis of PE, using ECG findings can be helpful in differentiation of massive PE from non-massive cases (15). 

T invert is another finding in V1-V4 leads in ECG, which was found in 23% of patients with PE. In a prospective study, it has been shown that inversion of T wave is the most common ECG abnormality in precordial leads of patients with PE, which correlates with severity and volume of the embolism (15, 16). In the study performed by Bircan et al. it was shown that nearly all of the abnormal ECG findings in PE patients were associated with severe PE (15).

It seems that although ECG findings do not have the required power for confirming or ruling out PE diagnosis, they can be helpful in triage of the patients and determining the severity of disease.

Regarding arterial blood gases, groups of patients with and without PE did not show a significant difference regarding arterial blood gas indices such as O_2_ pressure and CO_2_ pressure. These findings were in line with the results of the study carried out by Rodger et al. who showed that using arterial blood gas indices does not have the required ability to confirm or rule out PE diagnosis (17). This finding was also confirmed by other researchers such as Matsuoka et al. (18).

However, other researchers have shown that using arterial blood gas indices along with other diagnostic methods such as D-dimer has acceptable negative predictive value in ruling out PE diagnosis (17). Metafratzi et al. found a strong correlation between angiography obstruction and blood gas rates in PE patients. In the study, it was expressed that PaCO_2 _rate being 30 mmHg or lower was indicative of an obstruction index higher than 50% in the pulmonary arterial bed to a great extent (19). In another study, it was also shown that measuring arterial-alveolar O_2_ pressure slope is one of the simple and very useful methods for predicting short term prognosis in patients with acute PE (20). Masotti et al. also showed that there is a significant correlation between mortality rate among PE patients with decrease in O_2_ saturation and metabolic acidosis (21). 

In this study, the highest number of suspected PE cases belonged to internal medicine, surgery, and intensive care departments, respectively. In the study by Abidi et al. the highest number of suspected PE cases belonged to emergency, surgery, and intensive care departments (10). Based on the results of Salanci et al. study, most referred PE cases were from internal medicine and surgery departments, which is in line with the results of our study (22). 

Overall, although using clinical symptoms and ECG findings as well as evaluating arterial blood gas indices can help in diagnosis of PE in suspected patients, in our retrospective evaluation, no significant difference was found between the groups with and without PE in these regards. Further studies should be carried out with the aim of assessing the diagnostic value of these indices along with other clinical and laboratory methods.

## Limitations:

Having a retrospective design was among the limitations of the present study as it limited the ability to accurately assess the risk factors as well as the clinical symptoms in patients with suspected PE. Extraction of patients’ data from the history recorded in their profiles and not being able to evaluate the precision and accuracy of the recorded data was sometimes problematic for interpretation of the findings. Performing more comprehensive studies with prospective design and in multiple centers is suggested for more accurate evaluation of clinical and laboratory symptoms of patients with suspected PE.

## Conclusion:

Although attention to thrombosis risk factors, clinical symptoms, and laboratory findings, can be helpful in screening patients with suspected PE, considering the ability of CT scan in confirming or ruling out other possible differential diagnoses, it seems that a revision should be done to lower the threshold of ordering this diagnostic modality for suspected patients.
